# Patient characteristics and perceived health status of individuals with HIV and tuberculosis coinfection in Guangxi, China

**DOI:** 10.1097/MD.0000000000006475

**Published:** 2017-04-07

**Authors:** Yujia Zhu, Jizhou Wu, Xue Feng, Huanhuan Chen, Huaxiang Lu, Li Chen, Liuhong Luo, Chao Rui

**Affiliations:** a Department of Infection, Eighth Affiliated Hospital of Guangxi Medical University, Guigang City People's Hospital, Guigang; b Department of Infection, First Affiliated Hospital of Guangxi Medical University, Nanning, Guangxi, China; c School of Pharmacy, West Virginia University, Morgantown, WV, USA; d Guangxi Zhuang Autonomous Region Center for Disease Control and Prevention, Nanning; e Guangxi University of Chinese Medicine, Nanning, Guangxi, China.

**Keywords:** clinical profile, HIV and tuberculosis coinfection, HIV treatment, HIV/TB coinfected, medication profile, perceived health, self-efficacy, social support

## Abstract

To explore demographics, clinical and medication profiles, patients’ social support, and perceived health status in HIV/TB coinfected patients in Guangxi, China.

We performed a cross-sectional study in the HIV clinic of the Guigang City People's Hospital (N = 150). Health professionals conducted face-to-face interviews and collected data from patients’ electronic medical records regarding patients’ demographic, clinical, and medication information, as well as their social support and perceived health status. We classified all HIV/AIDS patients into HIV monoinfected and TB coinfected, at a ratio of 2:1.

Compared with the HIV monoinfected, patients with HIV/TB coinfection were more likely to be older, male, less educated, unemployed, carrying health insurance, having advanced stage of HIV infection, longer history with HIV, and other opportunistic infections. Patients coinfected with TB were also more likely to hold a negative belief that their HIV treatment could prevent exacerbations, and reported significantly worse emotional/informational support, social interaction, and perceived health status. Better social support and better self-efficacy to the HIV treatment adherence was significantly associated with better perceived health status among patients with HIV and TB coinfection.

Having HIV/TB coinfection was associated with poorer perceived general well-being and mental health, particularly in those undergoing TB therapy. Our findings suggest the need for mental health referrals and medication management for coinfected individuals, as well as further efforts and policies to improve coordinated care.

## 1 Introduction

Tuberculosis (TB) and HIV/AIDS are the 2 most common causes of death from infectious diseases across the world.^[1]^ In China, the total estimated number of people living with HIV/AIDS rose to 780,000 in 2011.^[2]^ The HIV pandemic has also led to an increase in the number of TB cases in the country such that, as of 2012, China had the 2nd highest TB burden in the world.^[3]^ Guangxi, a southern province of China, has recently experienced the HIV epidemic, and the prevalence of TB in Guangxi was 650 per 100,000 people.^[4,5]^ HIV/TB coinfection is one of the most common coinfections in this region, with a self-reported rate of 5.0% among HIV-infected individuals in a 2010 survey.^[5]^

In China, patients with HIV and TB coinfection can receive free antiretroviral therapy (ART) for HIV through the China Comprehensive AIDS Response Program (China).^[6]^ In general, patients can receive free TB care (ie, essential anti-TB medications, X-rays, and sputum checks) from the national TB programs.^[7]^ A patient generally obtains ART from the designated hospital near his or her residence, and TB medications from the local county-level Centers for Disease Control and Prevention (CDC) of China.^[8]^ According to World Health Organization (WHO) guidelines, the priority for HIV/TB coinfected patients is to take the TB treatment first, followed by ART for HIV within 8 weeks of starting TB treatment.^[9]^

On the other hand, the introduction of ART, and its success in treating HIV/AIDS patients, has significantly changed both the prognosis for patients and the role of HIV disease management as HIV infection has evolved over the past 2 decades into a chronic disease.^[10]^ The idea of HIV infection as a chronic disease brings with it unique health care needs (eg, lifelong therapy) and requires more attention to patients’ health status, as well as the psychosocial factors that might be associated with their treatment management, in particular for those with coinfection, for example, HIV/TB coinfection. Concurrent treatment of HIV and TB may induce drug–drug interactions between rifamycin and ART, and the risk of overlapping toxic effects and immune reconstitution inflammatory syndrome events may be enhanced.^[11–14]^ These may impact or be associated with not only patients’ clinical outcomes, but also their beliefs about the HIV treatment, self-efficacy to the HIV treatment adherence, and their health status.

Moreover, there is very limited research on the demographic characteristics, clinical profiles, psychosocial factors, and perceived health status in HIV/TB coinfected patients in China. Overall, within the context of China's high burden of both HIV and TB, particularly in areas experiencing HIV epidemics (eg, Guangxi), and also in light of the complexity of the current health care delivery system and ongoing health reform in China, we aimed to provide a delineation of the clinical and psychological profiles, as well as patients’ perceived health status in the HIV/TB coinfected population in Guangxi, China and compare them with those of HIV monoinfected patients.

## 2 Methods

### 2.1 Study design, setting, and population

We performed a cross-sectional study in the HIV/AIDS clinic of the Guigang City People's Hospital. Guigang City People's Hospital is a public hospital in Guigang City, which serves patients residing in Guigang City and nearby counties. Instrument and procedure of the study were reviewed and approved by the Institutional Review Board, Guangxi CDC, China.

The inclusion criteria were: over 18 years old; being infected by HIV, or coinfected with TB; and having taken current regimen of ART for at least 2 weeks. Our study excluded those who were: unable to take the survey because of cognitive dysfunction; hospitalized; and coinfected with hepatitis B virus or hepatitis C virus. We determined TB coinfection status through self-reported TB diagnosis with confirmation from medical records and self-reported receipt of TB treatment within the past 1 year. Participants with TB coinfection in this study either were undergoing TB treatment at the time of interview or had completed/stopped TB treatment within the past 2 months. Since the status of TB treatment was self-reported without medical records for verification, those who reported that they had completed or stopped TB treatment may not have been clinically TB-free. It is possible that these patients discontinued TB treatment by themselves. Besides, because of the frequent recurrence of TB, we considered those who had completed or stopped TB treatment within the past of 2 months to be TB coinfected in our analyses. We applied proportional sampling applied to recruit more HIV/TB coinfected patients to ensure enough power for the analysis. We classified eligible patients into HIV monoinfected and TB coinfected at a ratio of 2:1.

Between November 2015 and February 2016, trained health professionals approached potential participants during their routine visits to the HIV clinics at Guigang City People's Hospital. Health professionals obtained consent from eligible participants first. Then they conducted a face-to-face interview with each participant and administered a structured questionnaire that included questions about demographic, clinical, and psychosocial information, as well as the perceived health. The participation of subjects was voluntary, and participants could stop and refuse to answer the questions at any time. A stipend of 50 yuan ($8.50) was offered to each participant and interviewer as compensation per interview. Health professionals conducted all interviews in a private room in Guigang City People's Hospital. Other clinical information was obtained from patients’ medical records. We used EpiData 3.1 (The EpiData Association, Odense, Denmark) to enter the data.

### 2.2 Variable measurement

#### 2.2.1 Socio-demographic profile

Demographic information included gender (male, female), age, ethnicity (Han, the other minority), education level (below primary school, primary school, middle school, high school, and beyond high school), marital status (married, single, divorced, separated, and widowed), occupation (farmer, worker, other, and unemployed), annual income level (below Ren Min Bi [RMB] 10,000, between RMB 10,000 and 30,000, and above RMB 30,000), residency area (urban, rural), living (working) outside of Guigang area (yes, no), travel time for ART, and health insurance coverage (yes, no).

#### 2.2.2 Clinical profile

We collected medical records for the time since diagnosis of HIV, time since the initiation of ART, other opportunistic infections except TB, history of mental health illness (eg, schizophrenia, depression), the current ART regimen, and most updated WHO clinical staging of HIV/AIDS (WHO staging).^[15]^ We also asked participants whether they took traditional Chinese medicine for HIV (yes, no). In this study, other opportunistic infections were defined as and included pneumonia, shingles, thrush, and fungal and bacterial infections based on patients’ medical records.

### 2.3 Psychosocial factors

#### 2.3.1 Belief about taking the HIV treatment

We also asked the following 2 questions to assess patients’ belief about taking the HIV treatment, which included “Receiving HIV treatment (eg, the use of ART) can prevent the exacerbation of my HIV disease (in Chinese).” This items were scored on a 5-point Likert scale from “strongly disagree,” “disagree,” “neither agree nor disagree,” “agree,” and “strongly agree.”

#### 2.3.2 Self-efficacy to HIV treatment adherence

Patients’ self-efficacy to the HIV treatment adherence was measured by the HIV-Adherence Self-Efficacy Scale (HIV-ASES), which assesses HIV infected individual's confidence to perform important treatment-related behaviors in regard to adhering to treatment recommendations, which include but not limit to medication regimen, plans for nutrition, and exercise, in the face of barriers (permission to use the HIV-ASES was obtained from Dr Mallory Johnson, University of California, San Francisco).^[16]^ It has 12 items, each with a response scale ranging from 0 (“cannot do at all”) to 10 (“completely certain can do”). The total scores are between 0 and 120, with higher scores indicating higher perceived HIV-adherence self-efficacy. The HIV-ASES demonstrated robust internal consistency (rhos > 0.90) and good test–retest reliability among HIV-infected individuals.^[16]^

#### 2.3.3 Social support

We assessed social support for HIV patients using the Chinese version of the Medical Outcomes Study Social Support Survey (MOS-SSS-C), which includes 19 items.^[17]^ MOS-SSS-C is a psychometrically sound multidimensional measure to evaluate functional aspects of perceived social support across 4 different dimensions (emotional/informational, tangible, affectionate support, and positive social interaction). The survey includes 2 open-ended questions: “How many close relatives do you have?” and “How many close friends do you have?” The rest of the questions use a 5-point Likert scale. Each item is scored from “1” representing none of the time to “5” representing all of the time. Total scores of the scale and subscales range from 0 to 100, with higher scores indicating better perceived social support. Internal consistency (Cronbach α) was 0.97 for the overall MOS-SSS-C, and the 2-week test–retest reliability measured by intraclass correlation coefficients ranged from 0.74 to 0.84.^[17]^

### 2.4 Perceived health status

Perceived health status was assessed by 2 items that included “How do you rate your general health in the past month?” (on a 5-point scale, “Excellent,” “Very good,” “Good,” “Fair,” “Poor,” in Chinese), and “How often were you mentally at peace in the past month?” (on a 5-point scale, “All the time,” “Most of the time,” “Some of time,” “a little of time,” “None of time,” in Chinese). Higher scores indicated worse perceived health for both items.

### 2.5 Statistical analyses

All data were entered into the computer using EpiData 3.1 (The EpiData Association, Odense, Denmark). Exploratory factor analyses were performed for the HIV-ASES. The Cronbach α of the scale was obtained. Frequency and percentage of categorical variables were reported. Scores of each scale were presented. Chi square tests or Fisher exact tests were performed to analyze the association between each categorical independent variable and TB coinfection status. We conducted 2-sample *t* tests for continuous variables in the analyses in HIV/TB coinfected and HIV monoinfected patients. In the subgroup analyses, we used 2-sample *t* tests and Chi square tests (or Fisher exact test) for each combination of comparison on the psychosocial factors and perceived health, among the HIV monoinfected, the HIV/TB coinfected undergoing TB treatment, and the HIV/TB coinfected who had completed or stopped treatment in the past 2 months. We used SAS 9.3 (SAS Institute Inc., NC) for all the analyses in this study. We set the level of significance at *P* < 0.05 a priori.

## 3 Results

The response rate for the study was 150/194 (77.3%). Table 1 presents the descriptive statistics. The majority of the patients were male (103/150, 68.7%), married (112/150, 74.7%), of Han ethnicity (115/150, 76.6%), with education level of middle school or below (124/150, 82.7%), employed (111/150, 74%), living in rural areas (126/150, 84.0%), receiving an annual income below RMB 30,000 (around $4000) (81/117, 69.2%), living in big “Guigang” area (143/150, 95.3%), and carrying health insurance (84/150, 56%). The mean (standard deviation [SD]) age of the total sample was 44.31 (13.52). Of the 50 patients coinfected with TB, 58% were undergoing active TB treatment at the time of the interview, and 42% had completed or stopped TB treatment within the past 2 months. Most patients were treated with 1st-line ART (72.6%) in line with the Chinese HIV treatment guidelines. For these patients, 3TC + TDF + EFV (lamivudine + tenofovir + efavirenz) was the most frequently prescribed regimen (43.3%). A 2nd-line regimen, 3TC + TDF + LPV/r (lamivudine + tenofovir + lopinavir/ritonavir) accounted for 16.7% of the total ART prescriptions. Based on the factor loadings, we deleted item 3 in the HIV-ASES, since it was not closely related to the factors in the scale. Cronbach α coefficients was 0.93 for the HIV-ASES. The average scores of the HIV-ASES and the MOS-SSS-C were 97.8 (SD = 18.8) (range from 0 to 110) and 50.2 (SD = 19.8) (range from 0 to 100), respectively. Most of patients believed that their HIV disease progression would execrate if they did not receive the HIV treatment (78.7%, item score ≥4). A total of 31.3% of patients reported they were not mentally at peace most of time, and 64.0% of them rated their health status to be “poor” or “fair.”

**Table 1:**
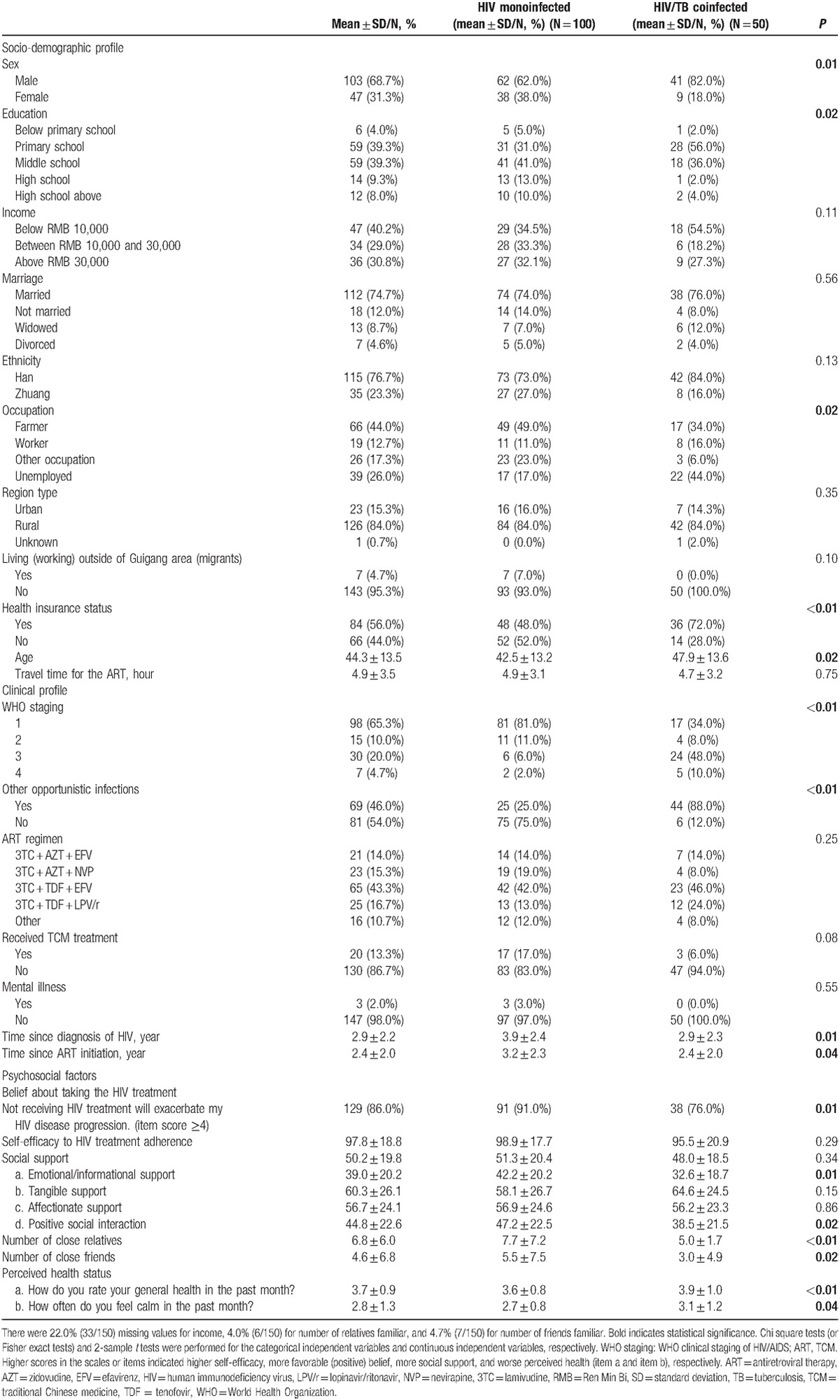
Factors associated with HIV/TB coinfection (N = 150).

Table 1 shows the characteristics of the HIV/TB coinfected population in comparison with patients with HIV monoinfection. Patients with HIV/TB coinfection were more likely to be male (*P* = 0.01), less educated (*P* = 0.02), unemployed (*P* = 0.02), older (*P* = 0.02), carrying health insurance (*P* < 0.01), have later WHO staging of HIV/AIDS (*P* < 0.01), have longer time since diagnosis of HIV (*P* = 0.01), have longer time since ART initiation (*P* = 0.04), and have other opportunistic infections (*P* < 0.01). Around 20% of the HIV monoinfected and 5% of the HIV/TB coinfected sought traditional Chinese medicine treatment for HIV, without statistically significant difference. No significant difference was found in ART regimen and self-efficacy to the HIV treatment between the HIV monoinfected and the HIV/TB coinfected. The HIV monoinfected were also more inclined to believe that the HIV treatment could prevent the exacerbation of their HIV disease (*P* = 0.01). Moreover, compared with the HIV monoinfected patients, those coinfected with TB scored worse on emotional/informational support (*P* = 0.01) and positive social interaction (*P* = 0.02). The HIV/TB coinfected patients also reported worse perceived health than those with HIV monoinfected in both items of “How do you rate your general health in the past month?” (*P* < 0.01) and “How often were you mentally at peace in the past month?” (*P* = 0.04).

Table 2 displays the subgroup analyses in patients with HIV monoinfection, HIV/TB coinfection with active TB treatment, and HIV/TB coinfection with recent completion of TB treatment. 1) HIV/TB co-infected patients who were undergoing active TB treatment reported significantly worse on their perceived health *(P* < 0.01) and were more inclined to hold a negative belief about taking the HIV treatment (*P* < 0.01) than those infected with HIV only. 2) Patients undergoing TB treatment also were more likely to report a negative belief about taking the HIV treatment than those who had just completed or stopped TB treatment. 3) HIV patients who had just completed or stopped TB treatment scored significant lower on the emotional/informational support (*P* < 0.01) and positive social interaction (*P* = 0.02) than those infected with HIV only.

**Table 2:**
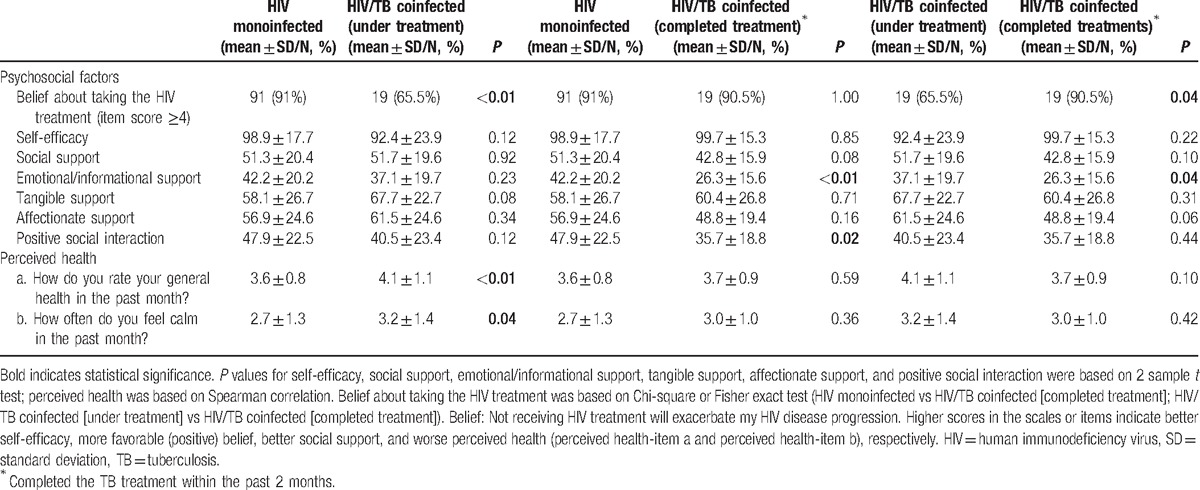
Subgroup analyses among HIV monoinfected (N = 100), HIV/TB coinfected under the active TB treatment (N = 29), and HIV/TB coinfected who completed the TB treatment within the past 2 months (N = 21).

Their scores on the emotional/informational support was also significant lower than those undergoing TB treatment (*P* = 0.04).

## 4 Discussion

With the increase of HIV and TB burden in China, it is important to have a clearer understanding of the potential health care needs of patients coinfected with HIV and TB. Our study is among the first to compare the demographic characteristics, clinical profiles, psychosocial factors, and perceived health between HIV monoinfected and HIV/TB coinfected patients in China. A thorough description of the characteristics in this population may help clinicians, policy-makers, and researchers assess patients’ medical needs, and it may identify potential targets for further clinical and policy strategies and interventions.

We found that HIV/TB coinfected individuals reported significantly worse perceived health. In our study, the coinfected population had more advanced stages of HIV/AIDS, potentially resulting in poorer physical well-being. Possibly, it is the severity of HIV progression that led to the worse of perceived health in patients coinfected with TB. Besides, the worse physical well-being of coinfected patients may be partially attributable to undergoing active TB treatment, which involves a high pill burden and greater risks of drug–drug interaction and toxicities associated with the comanagement of the 2 diseases. Thus, chronic viral infection such as TB seems to produce a diminished physical well-being among HIV/AIDS patients; however, the pathophysiology remains unclear.

On the other hand, HIV/TB coinfected patients were more likely to report not being mentally at peace, which indicated that those coinfected with TB might have increasing mental health care needs; however, only 3 patients in our sample had documented histories of mental illness. Under-diagnosis of mental health illness, such as posttraumatic stress disorder, major depression disorder, or anxiety, was probably common. It is likely that patients relied on their social support networks (eg, family, friends, or their significant others) for mental-health-related issues.^[18]^ In particular, patients coinfected with TB, particularly those completed or stopped their TB treatment within 2 months, were more likely to report lower scores on social interaction and emotional/informational support. In other words, these patients did not have enough support from people who could understand their situation nor people who could provide beneficial information to them. This finding is helpful for further programs aimed to improve the well-being of HIV-infected individuals, particularly those coinfected with TB, in this region. However, a substantial gap exists between the demands for mental care and supply of trained mental health professionals in China.^[19,20]^ Using screening tools for depression and anxiety as a component of routine HIV care may be one solution.^[18,21]^ Moreover, clinicians treating coinfected patients may need to increase their awareness of patients’ mental health, and make referrals to mental health services when necessary.^[19,22]^

According to our results, the HIV monoinfected and the HIV/TB coinfected differed in demographic backgrounds. Being older, male, less educated, unemployed, carrying health insurance, having advanced stage of HIV infection, longer history with HIV, and other opportunistic infections were more likely to have HIV and TB coinfection. Furthermore, many of those coinfected with HIV/TB (HIV/TB coinfected:HIV monoinfected = 24%:9%), particularly those undergoing the TB treatment did not hold a positive belief on their HIV treatment, which might be affected by their poor perceived health. In China's current health care system, the TB programs managed by the CDC take primary responsibility for prescribing and delivering the treatments for TB, while the general public hospitals take care of the HIV cases. The general hospitals have a limited role in the TB programs.^[5,7]^ Although the TB programs provide free care to TB patients and are cost-effective, the lack of technical support for the general hospitals from the TB program (eg, separate electronic medical records), as well as other contextual barriers (eg, lack of TB program funding) limit the collaboration between these 2 organizations.^[20,23]^ This may have a negative influence on coordinated care for HIV/TB patients. Such coordinated care may be of particular importance for HIV/TB comanagement given the complexity of the diseases and disease management as well as the poor perceived health among this population. Improving personnel training with more communication and technical support between these 2 organizations may help to reduce the barriers for coordinated care.^[5,20,23]^ Furthermore, a “designated hospital-based” model, integrating TB services in public hospitals, could be an alternative for HIV/TB coinfection management.^[22,23]^ In this model, a “designated” hospital provides the TB diagnosis and treatment, while the TB programs continue to provide the basic management; the evidence shows improvement in quality of care among TB patients in this model.^[21,22,24,25]^ Our findings in the differences in the population characteristics, clinical profiles, psychosocial factors, and perceived health status may provide evidence for future development to improve the HIV and TB coordinated care.

There are several limitations to this study. One major limitation is the small sample size in this pilot study. Given that around 5.0% prevalence of HIV and TB coinfection in this region and more than 1000 HIV patients responsible by the Guigang City People's Hospital,^[5]^ we designed to recruit around 50 HIV/TB coinfected patients, and our recruitment continued for 3 months. Although with small sample size, we were still able to find significant differences in demographic characteristics, perceived health, belief about taking the HIV treatment, and other psychosocial factors. Second, there may also be selection bias and reporting bias. For example, we excluded those hospitalized HIV patients, who were more likely to be in the advanced stage and coinfected with TB. Additionally, patients’ reports on TB treatment could be unreliable because of social desirability and recall bias. The results and conclusion may be more convincing if we could obtain more data for the analyses including verification of the duration of the TB treatment and more detailed clinical information regarding TB (eg, diagnoses of multidrug-resistant TB) and HIV (eg, cluster of differentiation 4 counts and viral loads at the time of interview). Since it was a cross-sectional study, we did not examine the causality and could not determine the temporal relationship of the exposure and the outcome. On the other hand, HIV/TB coinfection status was determined by the medical records and self-reported receipt of TB treatment, which could also lead to misclassification bias. It is possible that we did not capture all HIV patients with TB coinfection at the time of interview, and we were also not aware of the actual TB treatment status and patient's medication taking behavior. Another limitation is that the sample in this study may not well represent all Chinese HIV/TB coinfected patients. But most HIV-infected individuals living in Guigang City and nearby counties went to this hospital to see doctors for HIV. Therefore, this study may still provide a good representation of the HIV/TB coinfected population in central Guangxi.

In conclusion, HIV/TB coinfection in patients in Guangxi, China might be associated with poor health, which was more pronounced among those who were undergoing active TB therapy. Our findings may suggest the need for mental health referrals and medication management for the coinfected individuals. Additionally, further efforts and policies to improve coordination and collaboration between the general public hospitals and TB programs are warranted.
